# Supplementary material for: TAS-Seq is a robust and sensitive amplification method for bead-based scRNA-seq
Source: Commun Biol. 2022 Jun 27;5:602. doi: 10.1038/s42003-022-03536-0 (PMC9245575; doi:10.1038/s42003-022-03536-0)
Supplement: Supplementary file 2 — Supplementary Information [file 42003_2022_3536_MOESM2_ESM.pdf]

Supplementary Figure 1

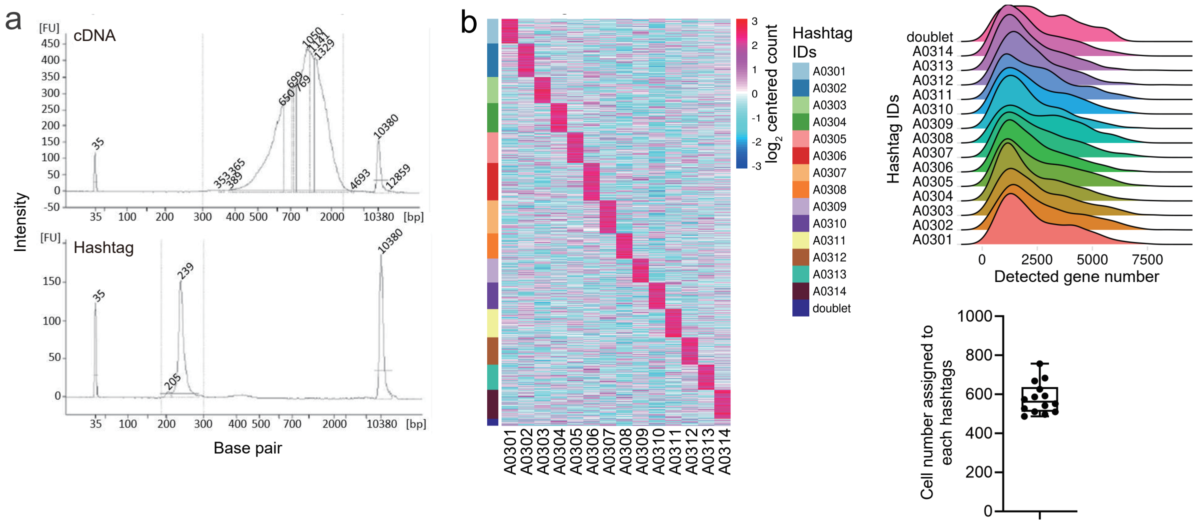

Supplementary Figure 1. TAS-Seq is compatible with cell hashing technology.

a. Size distribution of TAS-Seq amplified cDNA and hashtag libraries from CD45.2^{+} cells from a subcutaneous tumor model of Lewis lung carcinoma. **b.** Heatmap representation of normalized and log_2-centered hashtag count for each cell. Rows represents cells, and columns show each hashtag. Gene detection distribution and cell number of each hashtag-assigned sample are shown in the ridgeline and box plot. The box plot shows the mean of the cell number with upper and lower quantiles, and the whisker shows ±1.5×interquartile range. **a and b.** Representative results of two independent experiments are shown.

Supplementary Figure 2

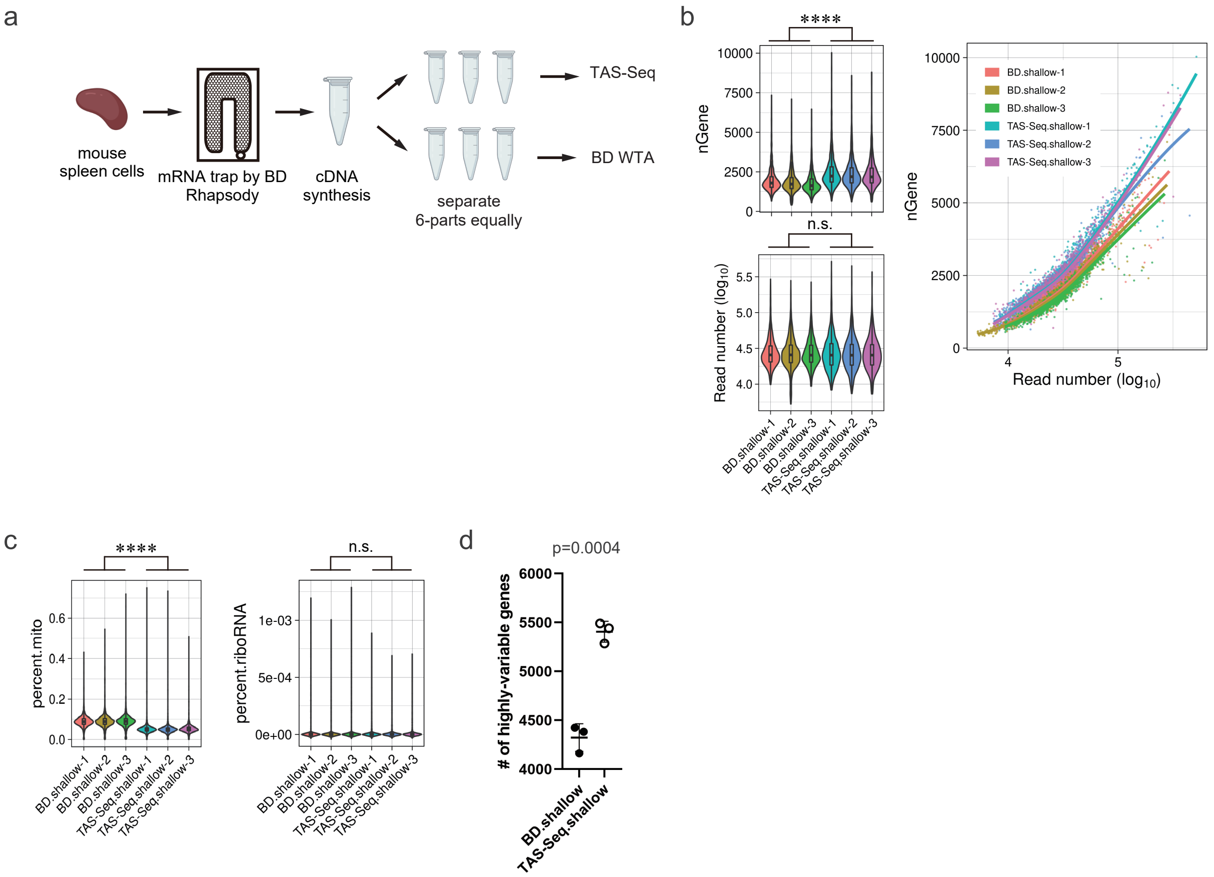

Supplementary Figure 2. TAS-Seq outperformed the BD Rhapsody WTA kit in terms of gene-detection sensitivity.
a. Diagram of the experimental workflow. Using fresh mouse adult spleen cells, cDNA synthesis was performed by the same BD Rhapsody cartridge, and resultant beads were separated into six parts. Three groups were processed by TAS-Seq, and the others were processed using a commercial BD WTA kit. **b.** Violin/box plot of read number and detected gene number, and scatter plot of read number/detected gene number, for each cell of TAS-Seq and BD WTA datasets. Box plot shows the mean of the read number with upper and lower quantiles. ****p = 0 (two-sided Wilcoxon rank-sum test). **c.** Violin/box plot of the composition of read number of mitochondrial genes and ribosomal RNA genes in TAS-Seq and BD WTA datasets of spleen cells. **d.** Number of highly-variable genes in TAS-Seq and BD WTA datasets of spleen cells. p=0.0004 by unpaired Student´s t-test (t=10.62, df=4). Error bars show mean ±SD in each sample. **b and c.** Exact p-values and statistics are shown in **Supplementary Data 5.** Box plot shows the mean of the read number with upper and lower quantiles, and the whisker shows ±1.5×interquartile range.

Supplementary Figure 3

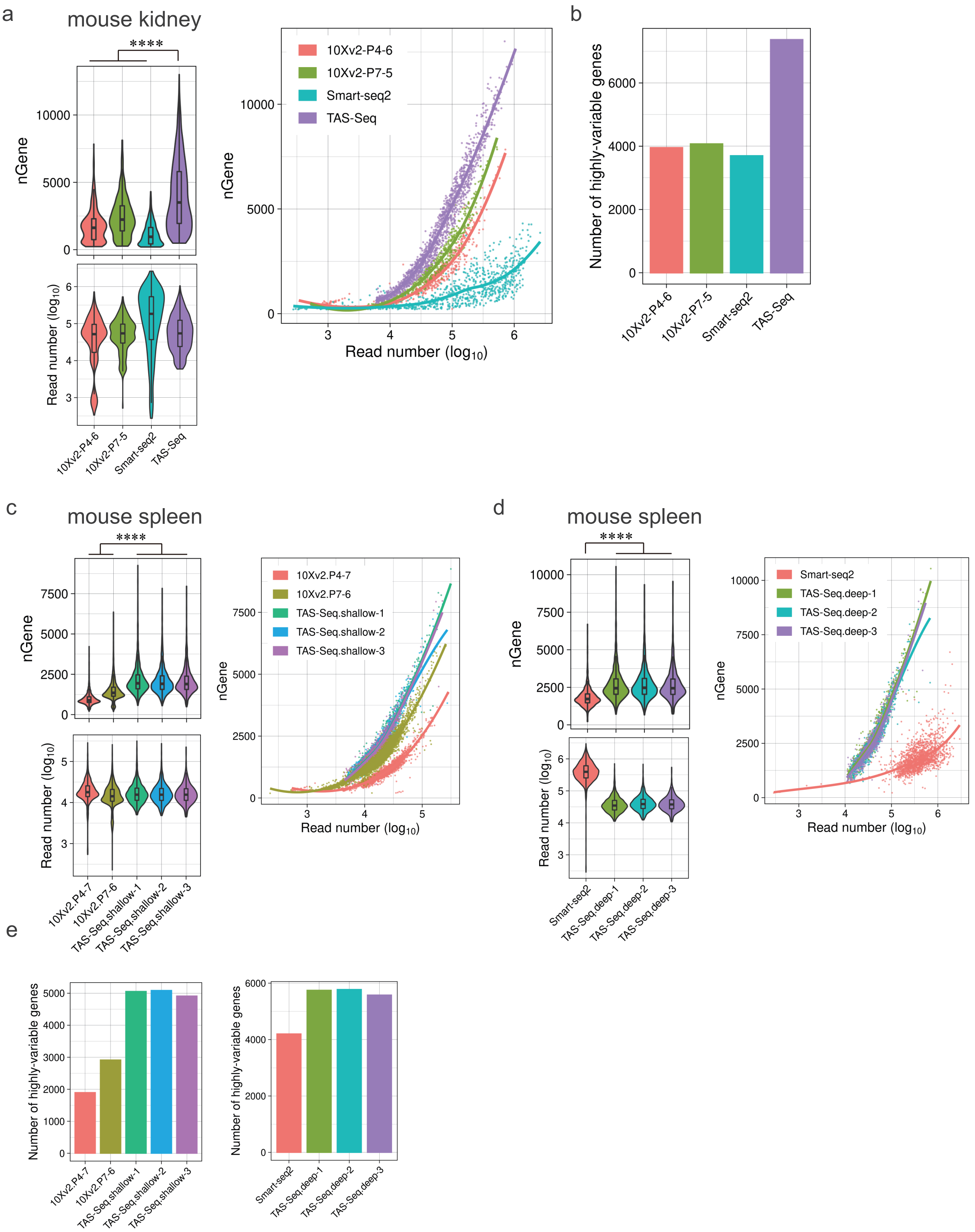

Supplementary Figure 3. TAS-Seq detects more overall and highly-variable genes than 10X v2 and Smart-seq2 in murine kidney and spleen tissue.

a, c and d. Violin/box plot of read number and detected gene number, and scatter plot of the read number/detected gene number, for each cell of TAS-Seq, 10X v2 (Tabula Muris), and Smart-seq2 datasets from murine kidney **(a)** or spleen **(c and d)** tissues. Box plot shows the mean of the read number with upper and lower quantiles, and the whisker shows ±1.5×interquartile range. ****p = 0 (two-sided Wilcoxon rank-sum test). **b and e.** Number of highly-variable genes in TAS-Seq, 10X v2 (Tabula Muris), and Smart-seq2 datasets in mouse kidney **(c)** or spleen **(e)** tissues. Exact p-values and statistics are shown in **Supplementary Data 5.**

Supplementary Figure 4

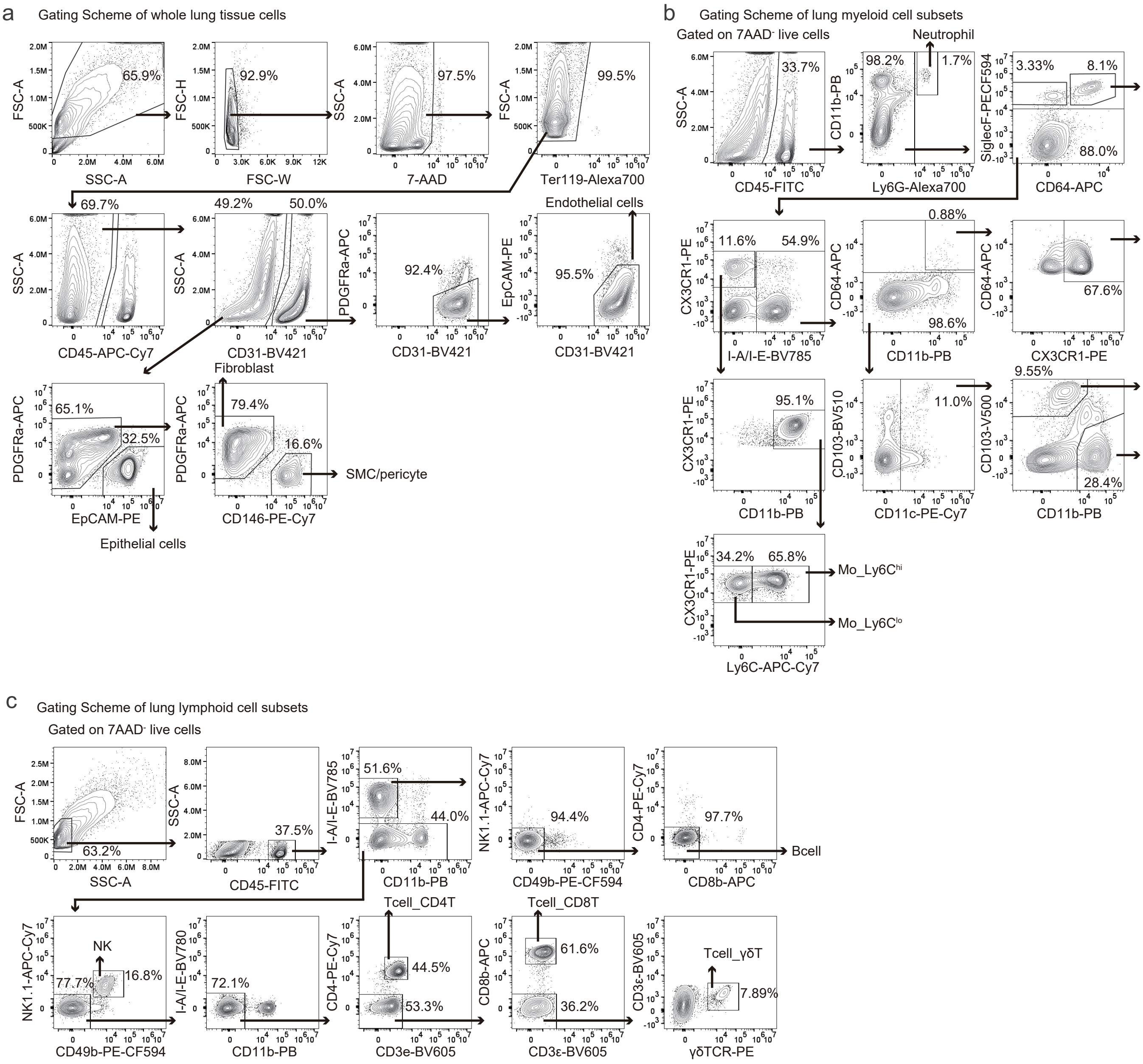

Supplementary Figure 4. Gating scheme for identification of murine lung cell subsets by flow cytometry.
Single-cell suspension of eight-week-old C57BL/6J female murine lung tissue was subjected to TAS-Seq analysis and analyzed by flow cytometry. **a.** Gating scheme for murine lung endothelial cells, epithelial cells, smooth muscle cells (SMC)/pericytes, and fibroblasts. **b.** Gating scheme for murine lung myeloid cell subsets. **c.** Gating scheme of murine lung lymphoid cell subsets.

Supplementary Figure 5

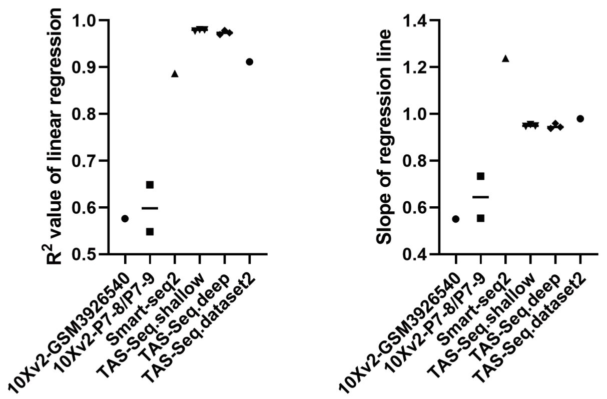

Supplementary Figure 5. Correlation analysis of cell composition between flow-cytometric data and scRNA-seq datasets of the murine lung.

Correlation analysis of cell composition between flow-cytometric data and scRNA-seq datasets of the murine lung. Pearson´s correlation coefficients and the slope of regression lines are shown.

Supplementary Figure 6

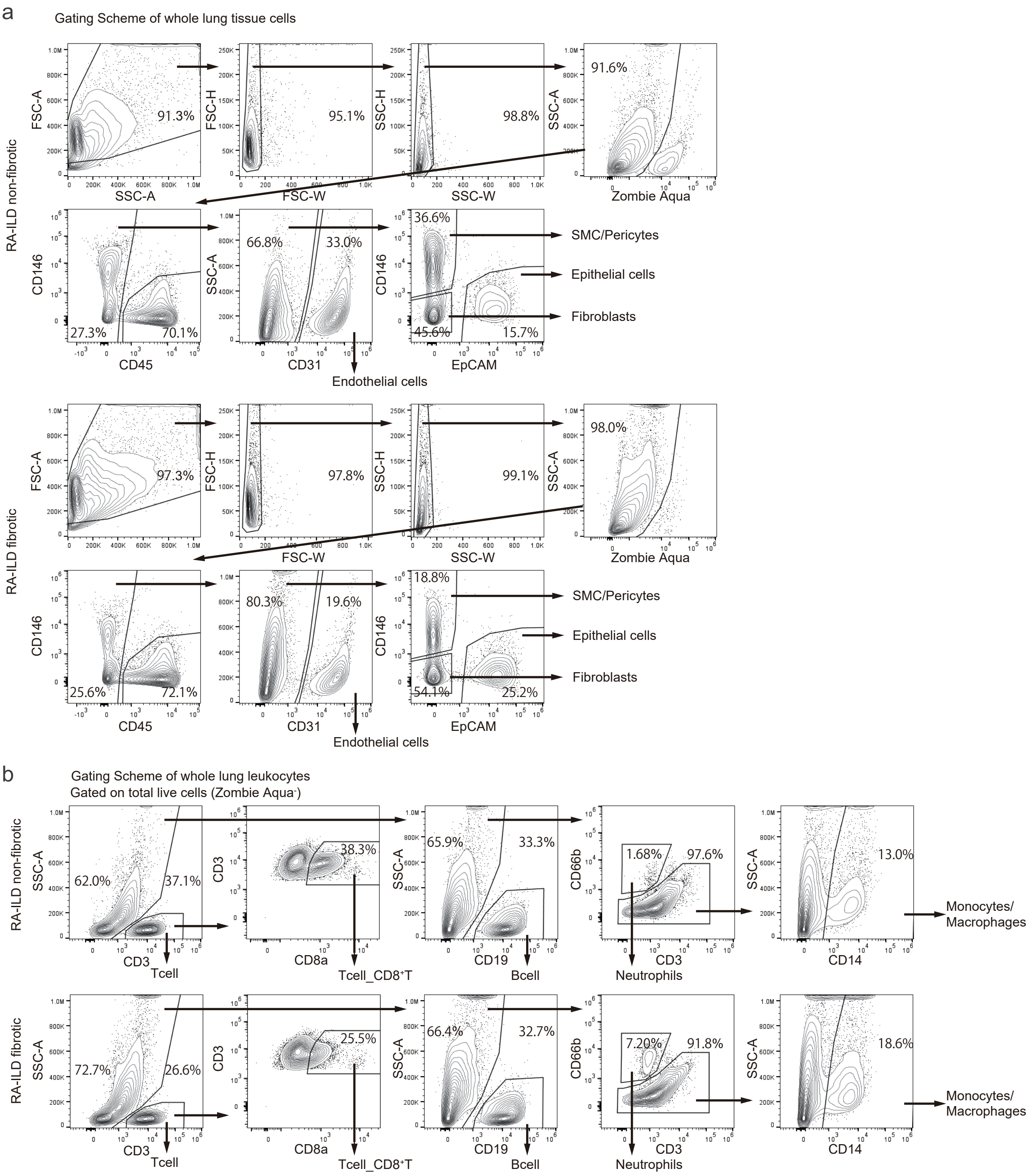

Supplementary Figure 6. Gating scheme for identification of human RA-ILD lung cell subsets by flow cytometry.

Single-cell suspension of non-fibrotic and fibrotic lung samples from a human RA-ILD patient was subjected to TAS-Seq analysis and analyzed by flow cytometry. **a.** Gating scheme for human lung endothelial cells, epithelial cells, smooth muscle cells (SMC)/pericytes, and fibroblasts. **b.** Gating scheme for human lung leukocytes.

Supplementary Figure 7

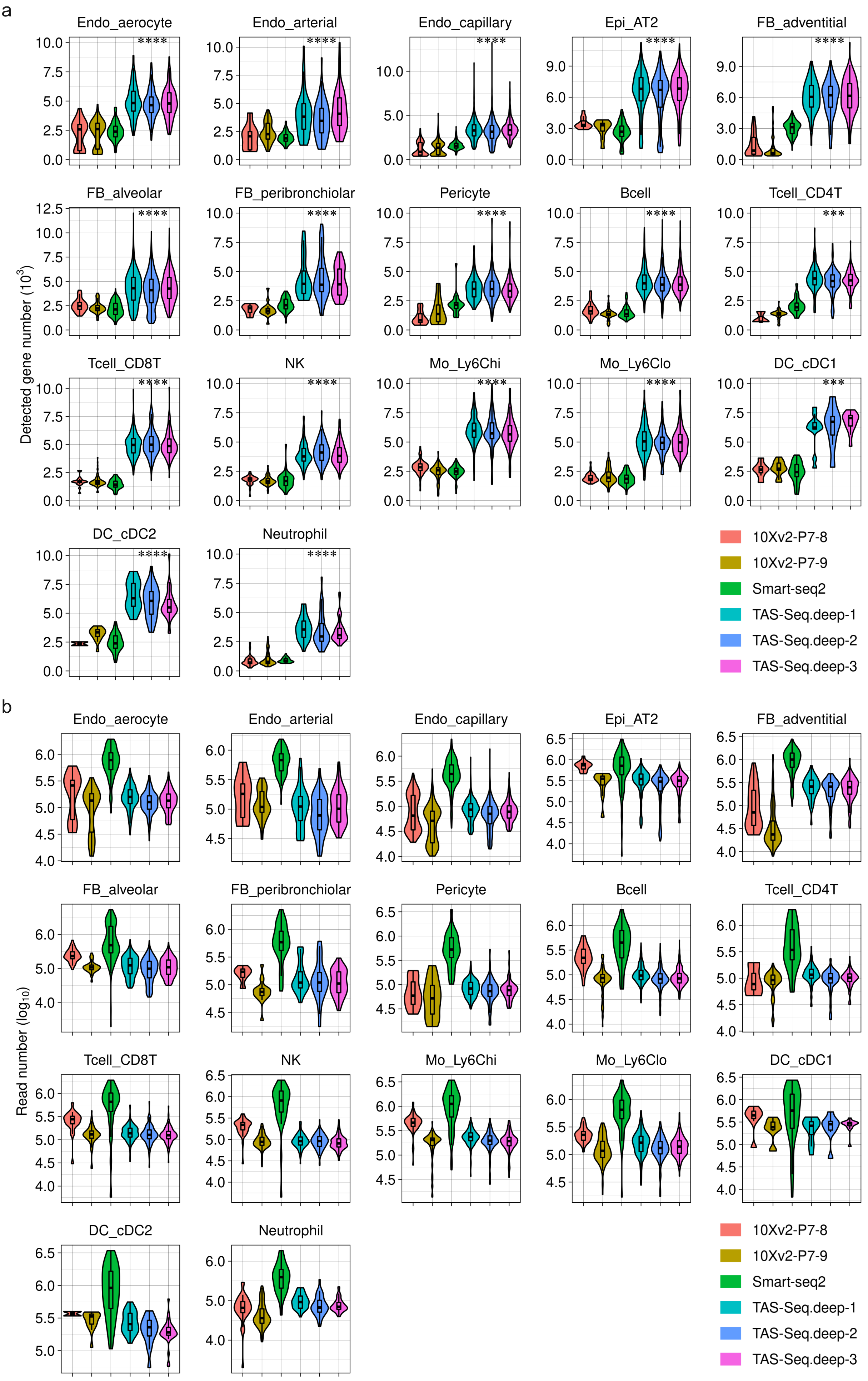

Supplementary Figure 7. Comparison of detected gene numbers in each cell subset of murine lungs between TAS-Seq, 10X v2 and Smart-seq2 datasets. **a.** Violin plot representing the distribution of detected gene numbers in each dataset among commonly-detected cell subsets for TAS-Seq, 10X v2 (Tabula Muris data), and Smart-seq2. Box plot shows mean, upper, and lower quantiles, and the whisker shows ±1.5×interquartile range of detected genes. ***p < 0.01, ****p < 0.001 by Wilcoxon rank-sum test when compared between TAS-Seq (deep-sequenced) and other datasets, apart from CD4 T cell and DC_cDC2 of 10Xv2-LungP7-8 and DC_cDC2 (p < 0.05 due to low number [<3] of these cells). Exact p-values and W statistics are shown in **Supplementary Data 5. b.** Violin plot representing the distribution of read number for each dataset among commonly-detected cell subsets in TAS-Seq, 10X v2 (Tabula Muris data), and Smart-seq2. Box plot shows mean, upper, and lower quantiles, and the whisker shows ±1.5×interquartile range of detected genes.

Supplementary Figure 8

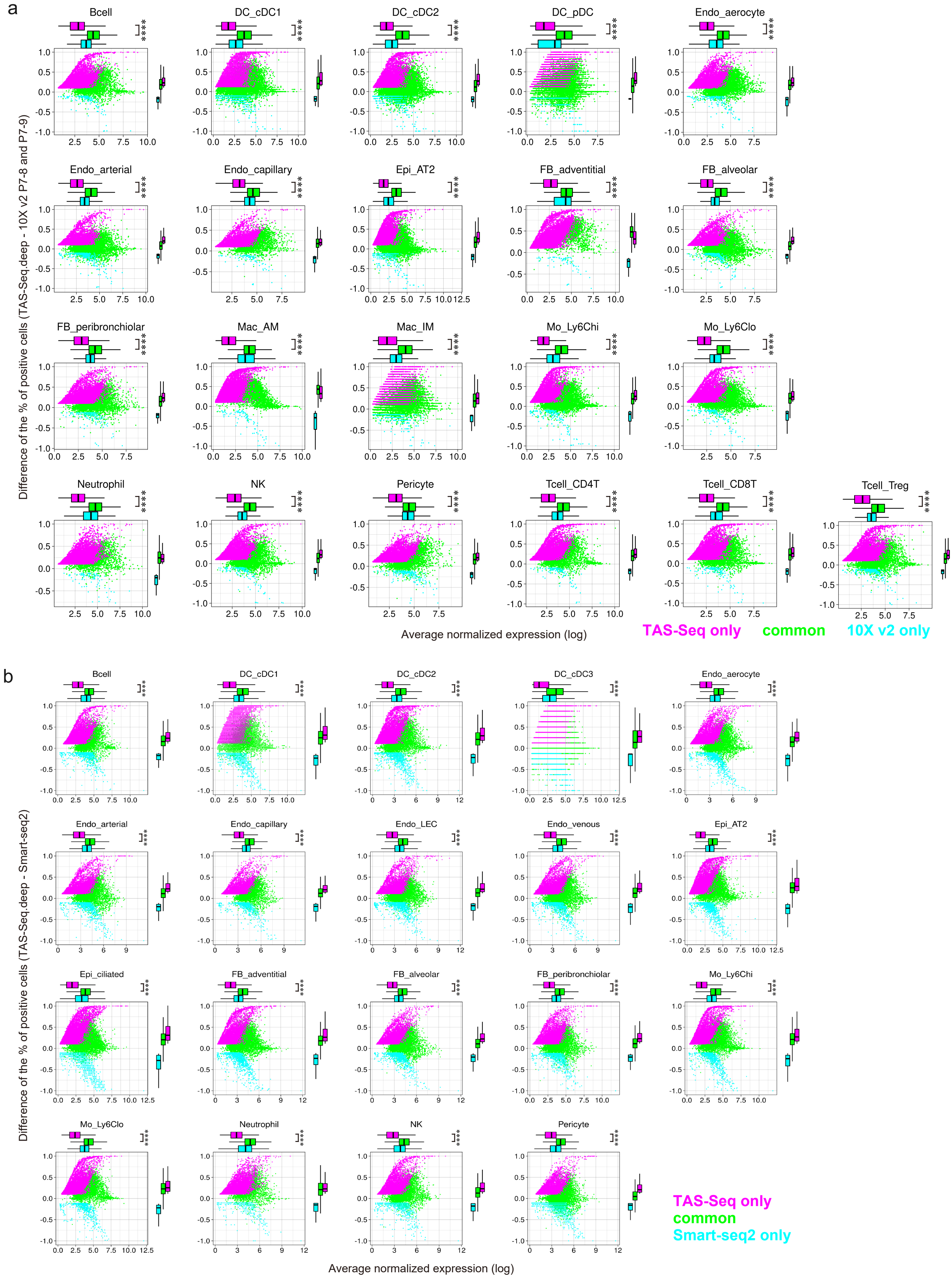

Supplementary Figure 8. Differences in drop-out rate of each gene in murine lung cell subsets among TAS-Seq, 10X v2, and Smart-seq2 datasets. a and b. Scatter plot of the average expression and difference in percentage of cells expressing each gene in each cell subset. Genes detected only in TAS-Seq, only in 10X v2, and commonly in both datasets are colored by magenta, cyan, and green, respectively **(a)**. Genes detected only in TAS-Seq, only in Smart-seq2, and commonly in both datasets are colored magenta, cyan, and green, respectively **(b)**. Upper boxplot shows mean, upper and lower quantiles, and the whisker shows ±1.5×interquartile range of the distribution of gene expression of each gene group; right boxplot shows mean, upper and lower quantiles, and the whisker shows ±1.5×interquartile range of the distribution of the difference of relative expression of each gene group. ****$p < 0.0001$ by Wilcoxon rank-sum test. Exact p-values statistics are shown in **Supplementary Data 5.**

Supplementary Figure 9

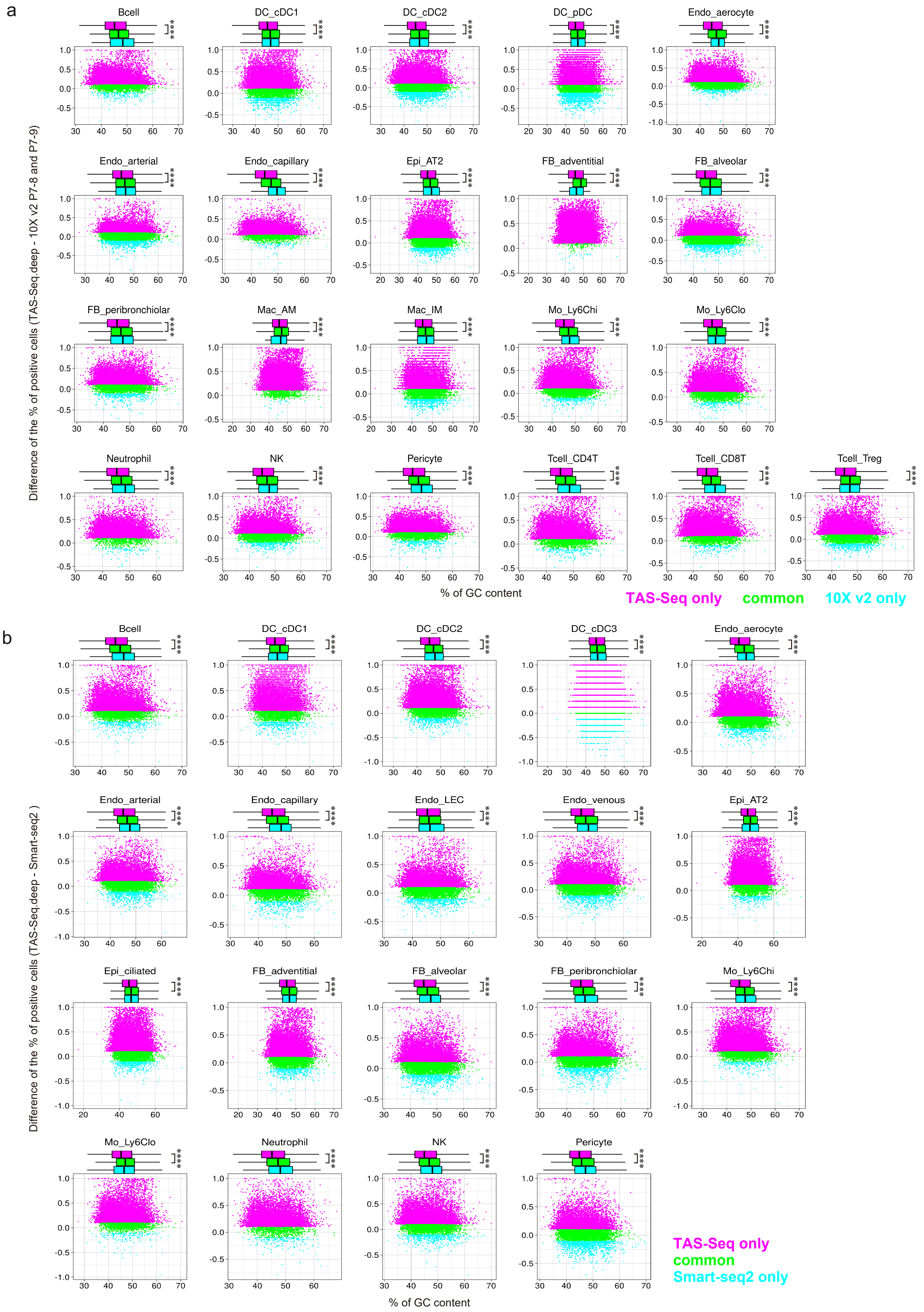

Supplementary Figure 9. Differences in percent GC content of each gene in murine lung cell subsets among TAS-Seq, 10X v2, and Smart-seq2 datasets. a and b. Scatter plot of percent GC content and the difference in percentage of cells expressing each gene in each cell subset. Genes detected only in TAS-Seq, only in 10X v2, and commonly in both datasets are colored magenta, cyan, and green, respectively **(a)**. Genes detected only in TAS-Seq, only in Smart-seq2, and commonly in both datasets are colored by magenta, cyan, and green, respectively **(b)**. Upper boxplot shows mean, upper and lower quantiles, and the whisker shows ±1.5×interquartile range of the distribution of percent GC content of each gene group. ****p < 0.0001 by Wilcoxon rank-sum test. Exact p-values and W statistics are shown in **Supplementary Data 5.**

Supplementary Figure 10

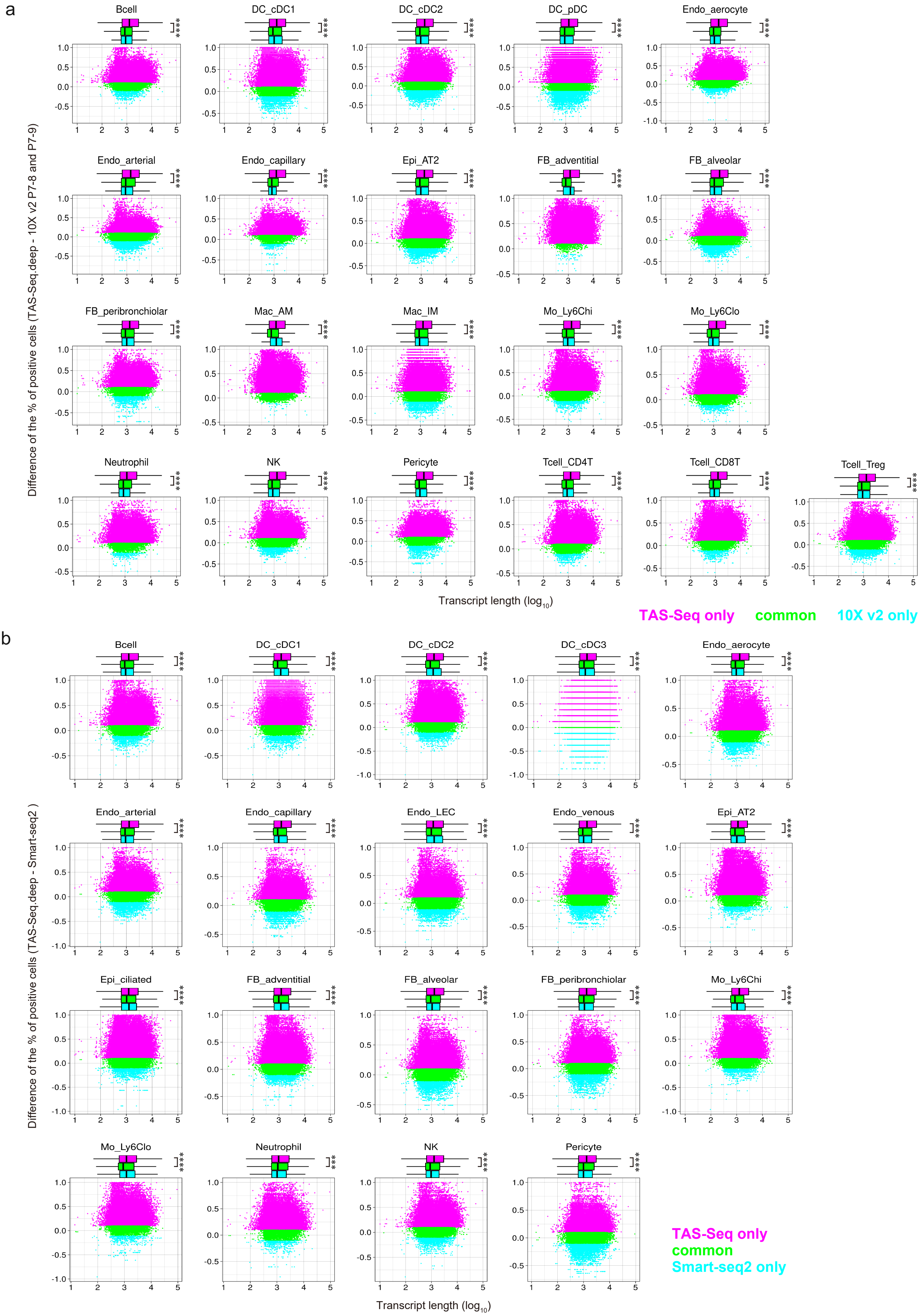

Supplementary Figure 10. Differences in length of each gene in murine lung cell subsets among TAS-Seq, 10X v2, and Smart-seq2 datasets. a and b. Scatter plot of transcript length and the difference in percentage of expressed cells for each gene in each cell subset. **a.** Genes detected only in TAS-Seq, only in 10X v2, and commonly in both datasets are colored magenta, cyan, and green, respectively. **b.** Genes detected only in TAS-Seq, only in Smart-seq2, and commonly in both datasets are colored by magenta, cyan, and green, respectively. Upper boxplot shows mean, upper and lower quantiles, and the whisker shows ±1.5×interquartile range of the transcript length distribution of each gene group. ****p < 0.0001, ***p < 0.001 by Wilcoxon rank-sum test. Exact p-values and W statistics are shown in **Supplementary Data 5.**

Supplementary Figure 11

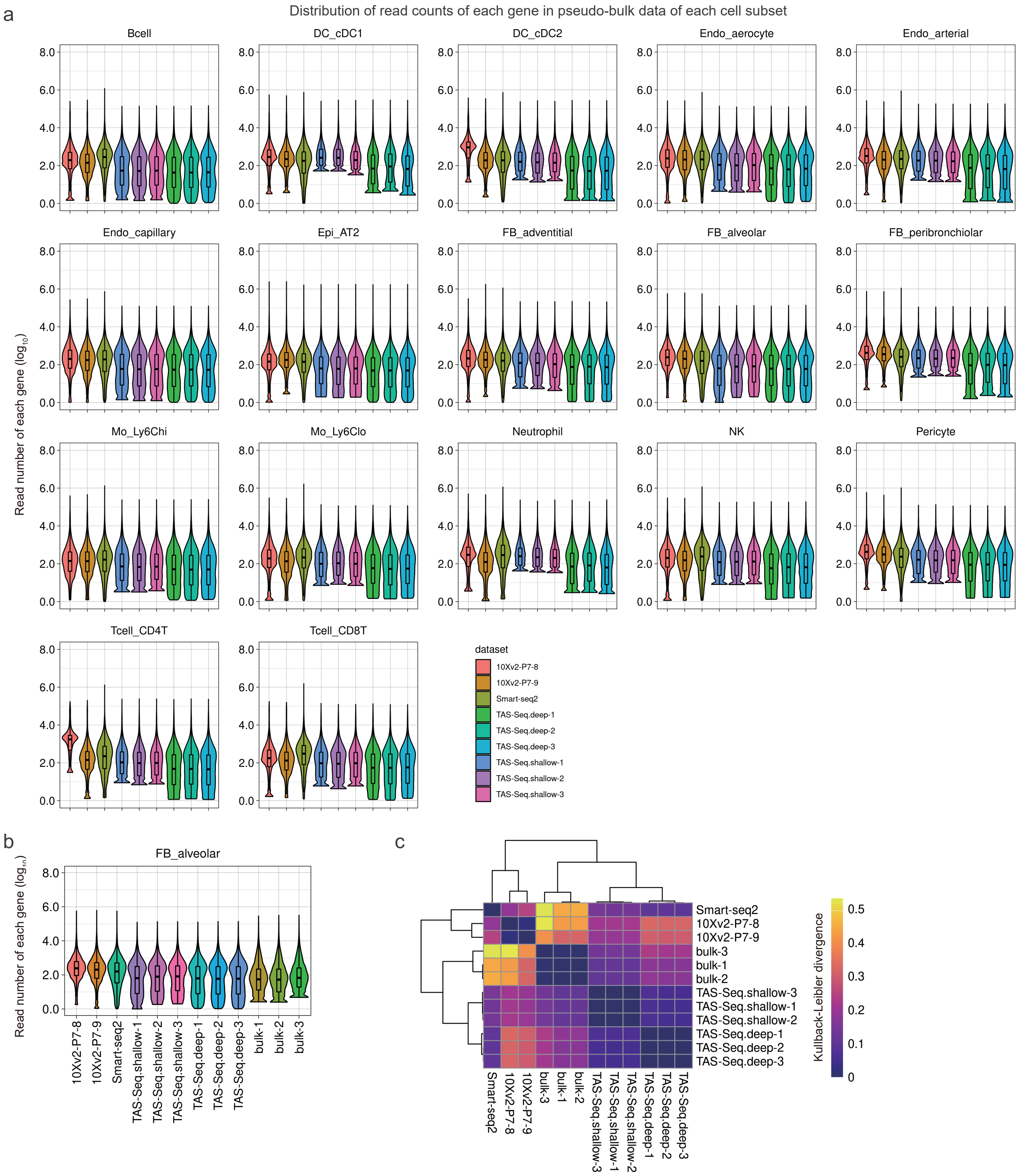

Supplementary Figure 11. Distribution of gene density by read number in pseudo-bulk data of each murine lung cell subset in TAS-Seq, Smart-seq2, and 10X v2 datasets.

a. Violin/box plot of gene density by read number in pseudo-bulk data of each murine lung cell subset in TAS-Seq, Smart-seq2, and 10X v2 datasets. Upper boxplot shows mean, upper and lower quantile, and the whisker shows ±1.5×interquartile range of the read number distribution. The total read number was normalized to 10,000,000 reads. **b.** Violin/box plot of gene density by read number in bulk RNA-seq data (bulk-1, bulk-2, bulk-3) of sorted murine lung fibroblasts (GSE110540, n=3). Pseudo-bulk data are also shown. Total read number of the datasets was normalized to 10,000,000 reads. Box plot shows mean, upper and lower quantile, and the whisker shows ±1.5×interquartile range of the read number distribution. **c.** Heatmap visualization of Kullback-Leibler divergence to evaluate the similarity distance among gene density results in bulk RNA-seq / pseudo-bulk data of murine lung fibroblasts.

Supplementary Figure 12

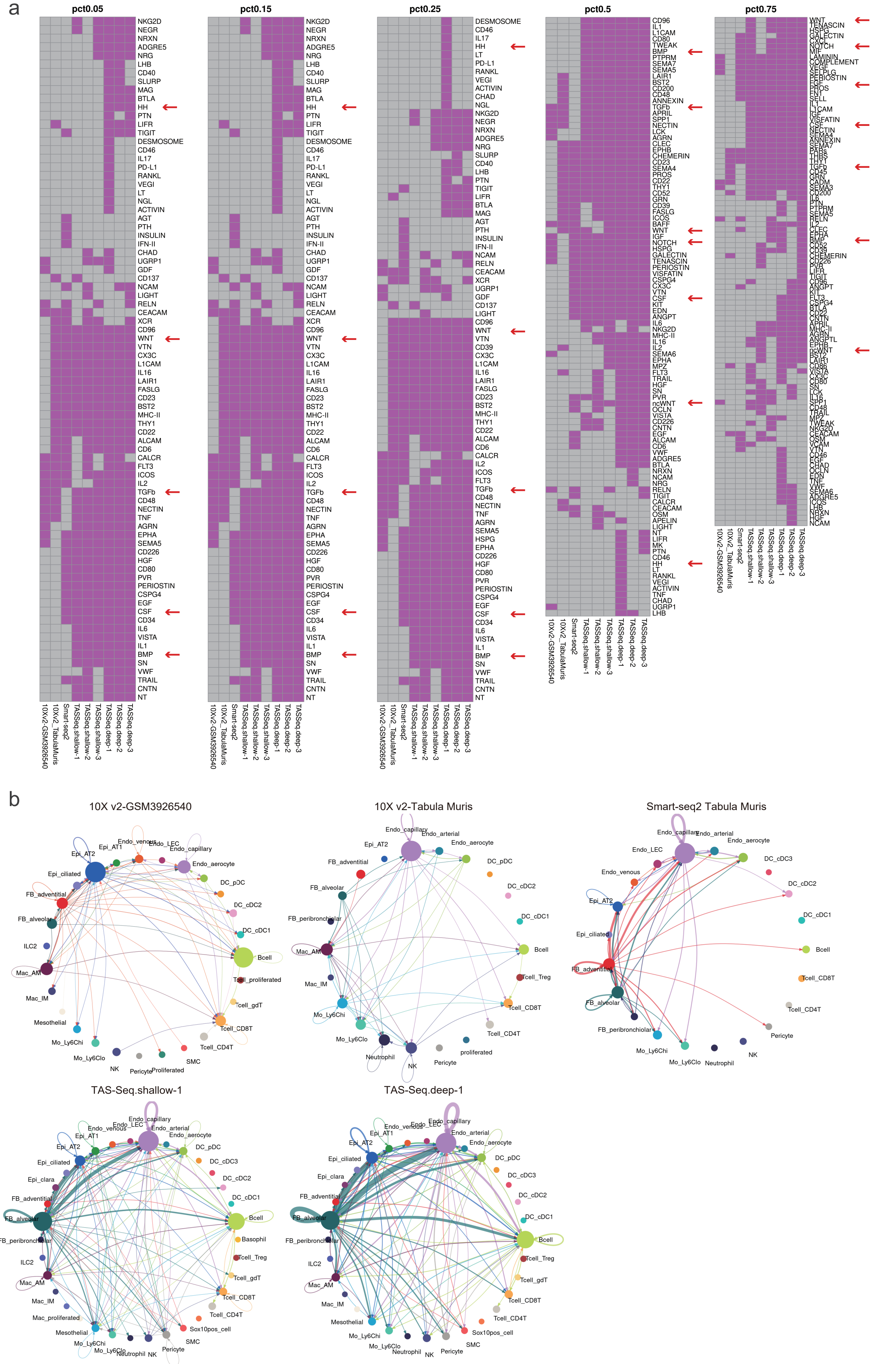

Supplementary Figure 12. Difference of CellChat-inferred Cell-cell interaction network of murine lungs between TAS-Seq, Smart-seq2, and 10X v2 datasets.

a. Heatmap representation of detected pathways within the network at several thresholds of minimum expression of genes in each cell subset (from 0.05 to 0.75). Detected pathways are colored by magenta, and undetected pathways are colored by grey. Commonly-detected pathways are separately shown in Supplementary Table 7 to show the difference between datasets. Fibroblast growth factor (FGF), bone morphologic protein (BMP), sonic hedgehog (HH), NOTCH, WNT, TGF, and CSF signaling are highlighted by red arrows. **b.** Circle plot visualizations of all of the cell-cell interaction networks of TAS-Seq, Smart-seq2, and 10X v2 datasets. Circle sizes are normalized to the cell number of each subset. Edge width represents communication strength (wider edge means stronger communication between source and target cell subsets), normalized among all datasets, and showed the top 10 percent of strong connections. Edge colors are the same as the color of their source cell subsets. Abbreviations of cell subsets were shown in **Supplementary Data 6.**
