# Supplementary material for: TAS-Seq is a robust and sensitive amplification method for bead-based scRNA-seq
Source: Commun Biol. 2022 Jun 27;5:602. doi: 10.1038/s42003-022-03536-0 (PMC9245575; doi:10.1038/s42003-022-03536-0)
Supplement: Supplementary file 3 — Description of Additional Supplementary Files [file 42003_2022_3536_MOESM3_ESM.pdf]

Description of Additional Supplementary Files

File name: Supplementary Data 1
Description: Cell annotations, associated marker genes, and associated references for mouse and human datasets.

File name: Supplementary Data 2
Description: List of the antibodies used for this study.

File name: Supplementary Data 3
Description: List of the primer sequences used for this study.

File name: Supplementary Data 4
Description: All of the identified marker genes of each dataset by Seurat analysis.

File name: Supplementary Data 5
Description: Statistics of Wilcoxon rank-sum test for Figure 3, Supplementary Figures 2, 3, 6, 7, 8, and 9.

File name: Supplementary Data 6.
Description: Abbreviations of the cell subset names.

File name: Supplementary Data 7
Description: List of commonly-detected pathways in CellChat analysis.

File name: Supplementary Data 8
Description: The source data behind the graphs (Figure 4d, 4h-i and 5a) in the paper
