# Supplementary material for: TAS-Seq is a robust and sensitive amplification method for bead-based scRNA-seq
Source: Commun Biol. 2022 Jun 27;5:602. doi: 10.1038/s42003-022-03536-0 (PMC9245575; doi:10.1038/s42003-022-03536-0)
Supplement: Supplementary file 5 — Reporting summary [file 42003_2022_3536_MOESM5_ESM.pdf]

nature research

Corresponding author(s): Kouji Matsushima
Last updated by author(s): May 27, 2022

# Reporting Summary

Nature Research wishes to improve the reproducibility of the work that we publish. This form provides structure for consistency and transparency in reporting. For further information on Nature Research policies, see our Editorial Policies and the Editorial Policy Checklist.

## Statistics

For all statistical analyses, confirm that the following items are present in the figure legend, table legend, main text, or Methods section.

| n/a | Confirmed | |
|---|---|---|
| ☐ | ☒ | The exact sample size ($n$) for each experimental group/condition, given as a discrete number and unit of measurement |
| ☐ | ☒ | A statement on whether measurements were taken from distinct samples or whether the same sample was measured repeatedly |
| ☐ | ☒ | The statistical test(s) used AND whether they are one- or two-sided<br>*Only common tests should be described solely by name; describe more complex techniques in the Methods section.* |
| ☒ | ☐ | A description of all covariates tested |
| ☐ | ☒ | A description of any assumptions or corrections, such as tests of normality and adjustment for multiple comparisons |
| ☐ | ☒ | A full description of the statistical parameters including central tendency (e.g. means) or other basic estimates (e.g. regression coefficient) AND variation (e.g. standard deviation) or associated estimates of uncertainty (e.g. confidence intervals) |
| ☐ | ☒ | For null hypothesis testing, the test statistic (e.g. $F$, $t$, $r$) with confidence intervals, effect sizes, degrees of freedom and $P$ value noted<br>*Give P values as exact values whenever suitable.* |
| ☒ | ☐ | For Bayesian analysis, information on the choice of priors and Markov chain Monte Carlo settings |
| ☒ | ☐ | For hierarchical and complex designs, identification of the appropriate level for tests and full reporting of outcomes |
| ☒ | ☐ | Estimates of effect sizes (e.g. Cohen's $d$, Pearson's $r$), indicating how they were calculated |

*Our web collection on statistics for biologists contains articles on many of the points above.*

## Software and code

Policy information about availability of computer code

**Data collection**

GNU wget 1.20.3 and sra-toolkit 2.11.3 was used for collect publicly-available data (Tabula Muris lung data).

**Data analysis**

For mapping analysis of this study, cutadapt 3.4, seqkit 0.15.0, bowtie2-2.4.2, mawk 1.3.4, GNU parallel 20190222, pigz 2.4, sed (GNU sed) 4.7, grep (GNU grep) 3.4, cat (GNU coreutils) 8.30, and ls (GNU coreutils) 8.30, CellRanger-6.1.2 were used other than R and Python packages in Ubuntu 20.04 LTS.

Python environment (pip) used for tis study is as follows.
Package Version
------------------- ---------
Package Version
----------------------- -------------------------
adjustText 0.7.3
altgraph 0.17.2
anndata 0.7.6
annoy 1.17.0
apturl 0.5.2
argon2-cffi 21.3.0
argon2-cffi-bindings 21.2.0
asciitree 0.3.3
attrs 19.3.0
Automat 0.8.0
backcall 0.2.0
bcrypt 3.1.7

BitVector 3.5.0
bleach 4.1.0
blinker 1.4
Brlapi 0.7.0
cellrank 1.5.1
certifi 2019.11.28
cffi 1.15.0
chardet 3.0.4
Click 7.0
cloud-init 22.1
cmake 3.21.2
colorama 0.4.3
command-not-found 0.3
configobj 5.0.6
constantly 15.1.0
cryptography 2.8
cupshelpers 1.0
cutadapt 3.4
cycler 0.10.0
Cython 0.29.19
dbus-python 1.2.16
debugpy 1.5.1
decorator 5.1.0
defer 1.0.6
defusedxml 0.7.1
demuxEM 0.1.7
distro 1.4.0
distro-info 0.23ubuntu1
dnaio 0.5.1
dnspython 1.16.0
docopt 0.6.2
docrep 0.3.2
docutils 0.17.1
dunamai 1.7.0
duplicity 0.8.12.0
entrypoints 0.3
fasteners 0.14.1
fbpca 1.0
fitsne 1.2.1
fonttools 4.28.5
forceatlas2-python 1.1
future 0.18.2
geosketch 1.2
get_version 3.5.3
GMM-Demux 0.2.1.3
gpg 1.13.1-unknown
gprofiler-official 1.0.0
h5py 3.4.0
harmony-pytorch 0.1.7
hnswlib 0.6.0
HTSeq 0.12.4
httplib2 0.14.0
hyperlink 19.0.0
idna 2.8
igraph 0.9.9
importlib-metadata 1.5.0
incremental 16.10.1
intervaltree 2.1.0
ipykernel 6.6.0
ipython 7.30.1
ipython-genutils 0.2.0
ipywidgets 7.6.5
isal 0.11.1
jedi 0.18.1
Jinja2 2.10.1
joblib 1.0.1
jsonpatch 1.22
jsonpointer 2.0
jsonschema 3.2.0
jupyter-client 7.1.0
jupyter-core 4.9.1
jupyterlab-pygments 0.1.2
jupyterlab-widgets 1.0.2
Keras 2.2.4
Keras-Applications 1.0.8
Keras-Preprocessing 1.1.2

keyring 18.0.1
kiwisolver 1.3.2
language-selector 0.1
launchpadlib 1.10.13
lazr.restfulclient 0.14.2
lazr.uri 1.0.3
legacy-api-wrap 1.2
Levenshtein 0.13.0
lightgbm 3.3.2
linalg 1.0.4
llvmlite 0.35.0
lockfile 0.12.2
loompy 3.0.6
louis 3.12.0
louvain 0.7.1
macaroonbakery 1.3.1
Mako 1.1.0
Markdown 3.1.1
MarkupSafe 2.0.1
matplotlib 3.5.1
matplotlib-inline 0.1.2
mistune 0.8.4
monotonic 1.5
more-itertools 4.2.0
mpi4py 3.1.3
natsort 7.1.1
nbclient 0.5.9
nbconvert 6.3.0
nbformat 5.1.3
nest-asyncio 1.5.4
netifaces 0.10.4
networkx 2.6.2
nmf-torch 0.1.1
notebook 6.4.6
numba 0.52.0
numcodecs 0.9.1
numexpr 2.7.3
numpy 1.20.0
numpy-groupies 0.9.13
oauthlib 3.1.0
olefile 0.46
packaging 21.3
pandas 1.3.0
pandocfilters 1.5.0
paramiko 2.6.0
parso 0.8.3
patsy 0.5.1
pegasusio 0.4.1
pegasuspy 1.4.4
petsc 3.16.1
pexpect 4.6.0
pickleshare 0.7.5
Pillow 8.4.0
pip 21.3.1
POT 0.8.1.0
progressbar2 4.0.0
prometheus-client 0.12.0
prompt-toolkit 3.0.24
protobuf 3.6.1
psutil 5.5.1
ptyprocess 0.7.0
pyasn1 0.4.2
pyasn1-modules 0.2.1
pybind11 2.9.0
pycairo 1.16.2
pycparser 2.21
pycrypto 2.6.1
pycups 1.9.73
pygam 0.8.0
Pygments 2.10.0
PyGObject 3.36.0
pygpcca 1.0.3
PyHamcrest 1.9.0
PyJWT 1.7.1
pymacaroons 0.13.0
PyNaCl 1.3.0

pynndescent 0.5.4
pyOpenSSL 19.0.0
pyparsing 2.4.6
pyRFC3339 1.1
pyrsistent 0.15.5
pysam 0.15.4
pyserial 3.4
python-apt 2.0.0+ubuntu0.20.4.7
python-dateutil 2.7.3
python-debian 0.1.36ubuntu1
python-igraph 0.9.9
python-utils 3.0.0
pytz 2019.3
pyxattr 0.6.1
pyxdg 0.26
PyYAML 5.3.1
pyzmq 22.3.0
regex 2021.8.28
reportlab 3.5.34
requests 2.22.0
requests-unixsocket 0.2.0
Rhapsody-python 0.0.1
scanorama 1.7.1
scanpy 1.8.2
scikit-learn 0.24.2
scikit-misc 0.1.4
scipy 1.7.0
screen-resolution-extra 0.0.0
scvelo 0.2.4
seaborn 0.11.2
SecretStorage 2.3.1
Send2Trash 1.8.0
service-identity 18.1.0
setuptools 45.2.0
setuptools-scm 6.3.2
simplejson 3.16.0
sinfo 0.3.4
six 1.14.0
sklearn 0.0
sortedcontainers 2.4.0
sos 4.3
ssh-import-id 5.10
statistics 1.0.3.5
statot 0.0.14
statsmodels 0.12.2
stdlib-list 0.8.0
subprocess32 3.5.4
systemd-python 234
tables 3.6.1
tabulate 0.8.9
terminado 0.12.1
testpath 0.5.0
texttable 1.6.4
threadpoolctl 2.2.0
tomli 2.0.0
torch 1.10.1
tornado 6.1
tqdm 4.62.2
traitlets 5.1.1
Twisted 18.9.0
typing-extensions 3.10.0.2
ubuntu-advantage-tools 27.6
ubuntu-drivers-common 0.0.0
ufw 0.36
umap-learn 0.4.6
unattended-upgrades 0.1
urllib3 1.25.8
usb-creator 0.3.7
velocyto 0.17.17
wadllib 1.3.3
wcwidth 0.2.5
webencodings 0.5.1
wheel 0.34.2
widgetsnbextension 3.5.2
wordcloud 1.8.1
wot 1.0.8.post3.dev8+gca5e94f

```
wrapt 1.13.3
xkit 0.0.0
xlrd 1.2.0
XlsxWriter 3.0.2
xopen 1.1.0
youtube-dl 2020.3.24
zarr 2.10.3
zipp 1.0.0
zope.interface 4.7.1


R version 4.1.3 (2022-03-10)
Platform: x86_64-pc-linux-gnu (64-bit)
Running under: Ubuntu 20.04.4 LTS

Matrix products: default
BLAS: /usr/lib/x86_64-linux-gnu/openblas-pthread/libblas.so.3
LAPACK: /usr/lib/x86_64-linux-gnu/openblas-pthread/liblapack.so.3

locale:
LC_CTYPE=en_US.UTF-8 LC_NUMERIC=C LC_TIME=en_US.UTF-8 LC_COLLATE=en_US.UTF-8 LC_MONETARY=en_US.UTF-8
LC_MESSAGES=en_US.UTF-8 LC_PAPER=en_US.UTF-8 LC_NAME=C LC_ADDRESS=C LC_TELEPHONE=C
LC_MEASUREMENT=en_US.UTF-8 LC_IDENTIFICATION=C

attached base packages:
stats4 parallel stats graphics grDevices utils datasets methods base

other attached packages:
qs_0.25.3 ggsci_2.9 SingleR_1.6.1 flashClust_1.01-2 WGCNA_1.70-3
fastcluster_1.2.3 dynamicTreeCut_1.63-1 parallelDist_0.2.6 cluster_2.1.2 DT_0.21
stringr_1.4.0 rmdformats_1.0.3 knitr_1.37 pheatmap_1.0.12 cowplot_1.1.1
ggpubr_0.4.0 ggplotify_0.1.0 factoextra_1.0.7 ggplot2_3.3.5 fastSave_0.1.0
doFuture_0.12.0 future.apply_1.8.1 future_1.24.0 MASS_7.3-55 recommenderlab_0.2-7
registry_0.5-1 proxy_0.4-26 arules_1.7-3 SeuratObject_4.0.4 Seurat_4.1.0
mclust_5.4.9 Matrix_1.4-0 tibble_3.1.6 dplyr_1.0.8 doParallel_1.0.17
iterators_1.0.14 foreach_1.5.2 data.table_1.14.2 slingshot_2.0.0 TrajectoryUtils_1.0.0
SingleCellExperiment_1.14.1 SummarizedExperiment_1.22.0 GenomicRanges_1.44.0 GenomeInfoDb_1.28.4 IRanges_2.26.0
S4Vectors_0.30.2 MatrixGenerics_1.4.3 matrixStats_0.61.0 princurve_2.1.6 tradeSeq_1.3.04
rDBEC_0.3.1 Biobase_2.52.0 BiocGenerics_0.38.0 BiocParallel_1.26.2 reticulate_1.24

loaded via a namespace (and not attached):
rsvd_1.0.5 Hmisc_4.6-0 ica_1.0-2 zinbwave_1.14.2 lmtest_0.9-39
rprojroot_2.0.2 crayon_1.5.0 spatstat.core_2.4-0 rhdf5filters_1.4.0 nlme_3.1-155
backports_1.4.1 qlcMatrix_0.9.7 impute_1.66.0 GOSemSim_2.18.1 rlang_1.0.1
XVector_0.32.0 ROCR_1.0-11 irlba_2.3.5 limma_3.48.3 phylobase_0.8.10
stringfish_0.15.5 bit64_4.0.5 glue_1.6.2 rngtools_1.5.2 sctransform_0.3.3
spatstat.sparse_2.1-0 AnnotationDbi_1.54.1 DOSE_3.18.3 spatstat.geom_2.3-2 VGAM_1.1-6
tidyselect_1.1.2 fitdistrplus_1.1-6 XML_3.99-0.9 tidyr_1.2.0 zoo_1.8-9
xtable_1.8-4 magrittr_2.0.2 evaluate_0.15 cli_3.2.0 zlibbioc_1.38.0
rstudioapi_0.13 doRNG_1.8.2 miniUI_0.1.1.1 rpart_4.1.16 fastmatch_1.1-3
locfdr_1.1-8 treeio_1.16.2 shiny_1.7.1 BiocSingular_1.8.1 xfun_0.29
tidygraph_1.2.0 KEGGREST_1.32.0 ggrepel_0.9.1 ape_5.6-1 listenv_0.8.0
Biostrings_2.60.2 png_0.1-7 withr_2.4.3 bitops_1.0-7 slam_0.1-50
ggforce_0.3.3 plyr_1.8.6 sparsesvd_0.2 RcppParallel_5.1.5 pillar_1.7.0
cachem_1.0.6 kernlab_0.9-29 DelayedMatrixStats_1.14.3 vctrs_0.3.8 ellipsis_0.3.2
generics_0.1.2 RApiSerialize_0.1.0 NMF_0.23.0 tools_4.1.3 foreign_0.8-82
rncl_0.8.4 munsell_0.5.0 tweenr_1.0.2 fgsea_1.18.0 DelayedArray_0.18.0
fastmap_1.1.0 HSMMSingleCell_1.12.0 compiler_4.1.3 abind_1.4-5 httpuv_1.6.5
pkgmaker_0.32.2 plotly_4.10.0 GenomeInfoDbData_1.2.6 gridExtra_2.3 edgeR_3.34.1
lattice_0.20-45 deldir_1.0-6 utf8_1.2.2 later_1.3.0 jsonlite_1.8.0
scales_1.1.1 docopt_0.7.1 ScaledMatrix_1.0.0 sparseMatrixStats_1.4.2 carData_3.0-5
tidytree_0.3.8 pbapply_1.5-0 genefilter_1.74.1 lazyeval_0.2.2 promises_1.2.0.1
car_3.0-12 latticeExtra_0.6-29 goftest_1.2-3 spatstat.utils_2.3-0 checkmate_2.0.0
rmarkdown_2.11 Rtsne_0.15 softImpute_1.4-1 uwot_0.1.11 igraph_1.2.11
HDF5Array_1.20.0 survival_3.2-13 yaml_2.3.5 DDRTree_0.1.5 htmltools_0.5.2
memoise_2.0.1 locfit_1.5-9.4 graphlayouts_0.8.0 here_1.0.1 viridisLite_0.4.0
digest_0.6.29 assertthat_0.2.1 mime_0.12 densityClust_0.3.1 RSQLite_2.2.10
yulab.utils_0.0.4 blob_1.2.2 RNeXML_2.4.6 preprocessCore_1.55.2 fastICA_1.2-3
splines_4.1.3 Formula_1.2-4 Rhdf5lib_1.14.2 RCurl_1.98-1.6 broom_0.7.12
monocle_2.20.0 hms_1.1.1 rhdf5_2.36.0 colorspace_2.0-3 base64enc_0.1-3
aplot_0.1.2 nnet_7.3-17 bookdown_0.24 Rcpp_1.0.8 RANN_2.6.1
enrichplot_1.12.3 fansi_1.0.2 parallelly_1.30.0 R6_2.5.1 grid_4.1.3
ggridges_0.5.3 lifecycle_1.0.1 ggsignif_0.6.3 recosystem_0.5 leiden_0.3.9
DO.db_2.9 howmany_0.3-1 qvalue_2.24.0 RcppAnnoy_0.0.19 RColorBrewer_1.1-2
htmlwidgets_1.5.4 beachmat_2.8.1 polyclip_1.10-0 purrr_0.3.4 shadowtext_0.1.1
gridGraphics_0.5-1 mgcv_1.8-39 globals_0.14.0 htmlTable_2.4.0 patchwork_1.1.1
```

spatstat.random_2.1-0 clusterExperiment_2.12.0 codetools_0.2-18 GO.db_3.13.0 FNN_1.1.3
prettyunits_1.1.1 gridBase_0.4-7 gtable_0.3.0 DBI_1.1.2 ggfun_0.0.5
tensor_1.5 httr_1.4.2 KernSmooth_2.23-20 stringi_1.7.6 progress_1.2.2
reshape2_1.4.4 farver_2.1.0 uuid_1.0-3 annotate_1.70.0 viridis_0.6.2
ggtree_3.0.4 xml2_1.3.3 combinat_0.0-8 BiocNeighbors_1.10.0 float_0.2-6
ade4_1.7-18 scattermore_0.8 bit_4.0.4 scatterpie_0.1.7 jpeg_0.1-9
spatstat.data_2.1-2 ggraph_2.0.5 pkgconfig_2.0.3 rstatix_0.7.0

For manuscripts utilizing custom algorithms or software that are central to the research but not yet described in published literature, software must be made available to editors and reviewers. We strongly encourage code deposition in a community repository (e.g. GitHub). See the Nature Research guidelines for submitting code & software for further information.

## Data

Policy information about availability of data

All manuscripts must include a data availability statement. This statement should provide the following information, where applicable:

- Accession codes, unique identifiers, or web links for publicly available datasets
- A list of figures that have associated raw data
- A description of any restrictions on data availability

Raw data, annotated gene-expression matrix, and associated metadata from these experiments have been deposited in the NCBI gene expression omnibus (GEO); accession GSE180149 and GSE200090. Public data used for this study is available at https://figshare.com/projects/Tabula_Muris_Transcriptomic_characterization_of_20_organs_and_tissues_from_Mus_musculus_at_single_cell_resolution/27733 (Tabula Muris Smart-seq2 data), GSE109774 (Tabula Muris 10X v2 data), and GSM3926540 (10X v2 shallow-sequenced murine lung data), https://www.nature.com/articles/s41586-020-2922-4#Sec33; supplementary table 2 (cell abundancy data of human lungs of 10X v2 and Smart-seq2 data). Fig 3b-e, 4b, 4f were generated by R code and uploaded in github repository(https://github.com/s-shichino1989/TASSeq-paper). Source data underlying Fig 4d, 4h-I and 5a uploaded in github repository(https://github.com/s-shichino1989/TASSeq-paper) as Supplementary Data 8.

# Field-specific reporting

Please select the one below that is the best fit for your research. If you are not sure, read the appropriate sections before making your selection.

☒ Life sciences ☐ Behavioural & social sciences ☐ Ecological, evolutionary & environmental sciences

For a reference copy of the document with all sections, see nature.com/documents/nr-reporting-summary-flat.pdf

# Life sciences study design

All studies must disclose on these points even when the disclosure is negative.

| | |
|---|---|
| Sample size | No sample-size calculation was performed. |
| Data exclusions | No data were excluded from the analyses. |
| Replication | Findings from publicly-avaiable data were not replicated because publicly-avaiable data is unique one. |
| Randomization | n/a |
| Blinding | n/a |

# Reporting for specific materials, systems and methods

We require information from authors about some types of materials, experimental systems and methods used in many studies. Here, indicate whether each material, system or method listed is relevant to your study. If you are not sure if a list item applies to your research, read the appropriate section before selecting a response.

## Materials & experimental systems

| n/a | Involved in the study | |
|---|---|---|
| ☐ | ☒ | Antibodies |
| ☐ | ☒ | Eukaryotic cell lines |
| ☒ | ☐ | Palaeontology and archaeology |
| ☐ | ☒ | Animals and other organisms |
| ☐ | ☒ | Human research participants |
| ☒ | ☐ | Clinical data |
| ☒ | ☐ | Dual use research of concern |

## Methods

| n/a | Involved in the study | |
|---|---|---|
| ☒ | ☐ | ChIP-seq |
| ☐ | ☒ | Flow cytometry |
| ☒ | ☐ | MRI-based neuroimaging |

## Antibodies

| | |
|---|---|
| Antibodies used | A detailed list of used antibodies in this study is shown in Supplementary Table 2. |
| Validation | All of the antibodies used in this study were validated by the manufactures (BD Biosciences or BioLegend). |

## Eukaryotic cell lines

Policy information about cell lines

| | |
|---|---|
| Cell line source(s) | NIH/3T3 cells were purchased from ATCC(American Type Culture Collection). |
| Authentication | None of the cell lines used were authenticated. |
| Mycoplasma contamination | All cell lines used in this study tested negative for mycoplasma contamination. |
| Commonly misidentified lines (See ICLAC register) | n/a |

## Animals and other organisms

Policy information about studies involving animals; ARRIVE guidelines recommended for reporting animal research

| | |
|---|---|
| Laboratory animals | C57BL/6J female mice were purchased from Sankyo Labo Service Corporation (Ibaragi, Japan). All mice were bred at specific pathogen-free facilities at Tokyo University of Science and were 8 weeks old (for lung, spleen, and kidney samples) at the commencement of experiments. |
| Wild animals | The study did not involve wild animals. |
| Field-collected samples | The study did not involve sampled collected from the field. |
| Ethics oversight | All animal experiments were reviewed and approved by the Animal Experiment Committee of Tokyo University of Science (approval number: S17034, S18029, S19024, and S20019). |

Note that full information on the approval of the study protocol must also be provided in the manuscript.

## Human research participants

Policy information about studies involving human research participants

| | |
|---|---|
| Population characteristics | *Describe the covariate-relevant population characteristics of the human research participants (e.g. age, gender, genotypic information, past and current diagnosis and treatment categories). If you filled out the behavioural & social sciences study design questions and have nothing to add here, write "See above."* |
| Recruitment | Human lung samples were collected from lung cancer patients with pulmonary fibrosis who underwent curative surgical resection from August 2015 to December 2019 at Nara Medical University Hospital. Informed consent was obtained from all patients who participated in the study herein. Any of the recruitment bias might not be affected to the results of this study. |
| Ethics oversight | All human studies were approved by the Ethics Committee of Nara Medical University (Approval No. 1973) and Tokyo University of Science (Approval No. 18018). |

Note that full information on the approval of the study protocol must also be provided in the manuscript.

## Flow Cytometry

### Plots

Confirm that:

- [x] The axis labels state the marker and fluorochrome used (e.g. CD4-FITC).
- [x] The axis scales are clearly visible. Include numbers along axes only for bottom left plot of group (a 'group' is an analysis of identical markers).
- [x] All plots are contour plots with outliers or pseudocolor plots.
- [x] A numerical value for number of cells or percentage (with statistics) is provided.

### Methodology

| | |
|---|---|
| Sample preparation | For murine lung cells, single-cell suspensions were blocked with Fc block (anti-CD16/32, clone: 2.4G2, BioXcell, West Lebanon, NH, USA) and stained with appropriate antibody mixtures diluted with PBS supplemented with 2% FBS. After |

washing with PBS supplemented with 2% FBS, cells were suspended with PBS supplemented with 2% FBS and 0.5 µg/ml propidium iodide. For human lung cells, single-cell suspensions were washed once with PBS and stained with LIVE/DEAD Fixable Aqua Dead Cell Stain Kit (Thermo Fisher Scientific) at 4ºC for 30 minutes. Cells were washed once with PBS supplemented with 2% FBS; cells were blocked with 2% normal mouse serum and stained with appropriate antibody mixtures diluted with PBS supplemented with 2% FBS. After washing with PBS supplemented with 2% FBS, cells were suspended with PBS supplemented with 2% FBS. Data were collected on a Gallios flow cytometer and analyzed using FlowJo software v10.6.2 (BD Biosciences). A detailed list of used antibodies is shown in Supplementary Table 2.

| | |
|---|---|
| Instrument | Data were collected on a Gallios flow cytometer (Beckman Coulter) or a CytoFLEX S flow cytometer (Beckman Coulter). |
| Software | Data were analyzed using FlowJo software v10.6.2 (BD Biosciences). |
| Cell population abundance | n/a |
| Gating strategy | All of the gating strategy was shown in Supplementary Figure 4 and 5. |

☒ Tick this box to confirm that a figure exemplifying the gating strategy is provided in the Supplementary Information.
